# Extraskeletal Chondromatosis in a 30-Year-Old Patient: A Rare Case Report

**DOI:** 10.7759/cureus.65844

**Published:** 2024-07-31

**Authors:** Yaminy Ingale, Vidya Viswanathan, Arpana Dharwadkar, Nikita G Chhablani

**Affiliations:** 1 Pathology, Dr. D. Y. Patil Medical College, Hospital and Research Centre, Dr. D. Y. Patil Vidyapeeth (Deemed to be University), Pune, IND

**Keywords:** index finger, vimentin, immunohistochemistry, hyaline cartilage, extraskeletal chondroma

## Abstract

The term chondroma refers to a slow-growing benign tumor. When the tumor arises from the medullary cavity, it is referred to as enchondroma, which is a very common bone tumor. However, if it arises from soft tissues, which is extremely rare, it is referred to as soft tissue chondroma or extraskeletal chondroma. Extraskeletal chondromas are uncommon; benign soft tissue tumors that mostly originate from hyaline cartilage are unrelated to the periosteum, tendon, or bone. The most common sites include fingers and toes. The frequent presentation is a slow-growing, firm, painless, and occasionally tender soft tissue mass. Morphologically, it exhibits lobular structures of hyaline cartilage, and hence it becomes difficult to differentiate it from low-grade chondrosarcoma, so the alarming sign of differentiation becomes a must. Recurrence is possible if it is incompletely removed. Complete removal with the capsule is a must to avoid recurrence. Immunohistochemistry remains the cornerstone for a definite diagnosis when S100 protein and vimentin show positivity for tumor cells and the proliferation index (Ki67%) is low. In this study, we present a very uncommon case of a 30-year-old patient with soft tissue chondromatosis of the palmer aspect of the index finger and palm.

## Introduction

Extraskeletal chondroma is a rare benign cartilaginous soft tissue tumor lacking continuity to the periosteum or bone cortex [1]. The fingers and toes are where it typically manifests [2]. Extraskeletal chondroma was defined by Enzinger (1983). The hands and feet account for 90% of soft tissue chondroma cases [3]. The tumor is well defined and oval in shape; its diameter is rarely greater than 3 cm. In this case, it manifests as a tumor with various hand and finger symptoms. Since Baumuller's initial report in 1883, about 200 cases have been documented [4]. Moreover, extraskeletal chondroma in the dura mater, throat, mouth, and skin are infrequently observed. This tumor is frequently observed in middle-aged groups without gender differences [5]. A soft tissue mass that grows slowly, is painless, and occasionally painful are its common presentation. When these tumors are discovered, the majority of them are small. It can be because of their preferred spot. This is a rare case of 30-year-old patient with extraskeletal chondromatosis affecting the palm and index finger.

## Case presentation

**Figure 1:**
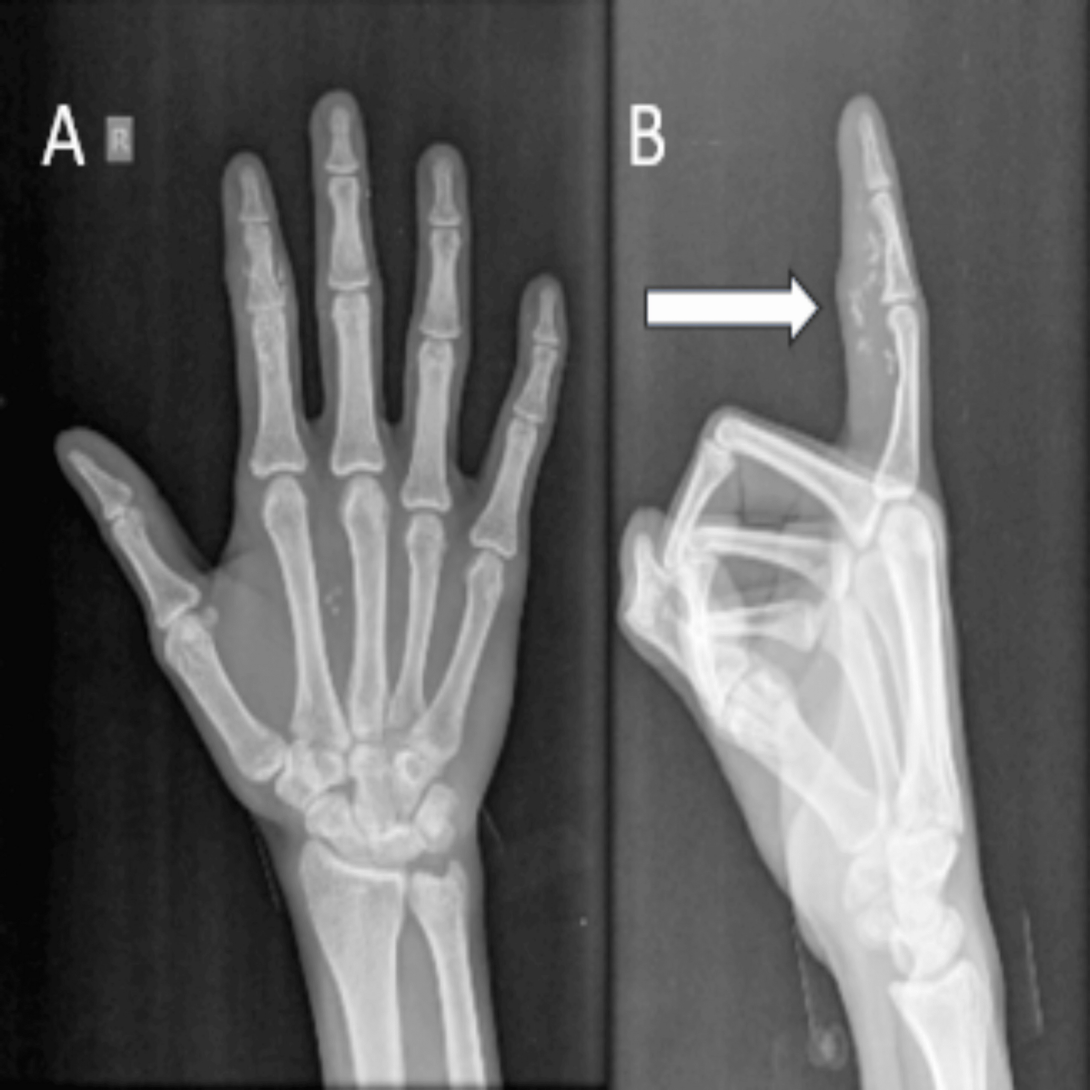
X-ray image of the right hand: (A) anteroposterior view of the right hand showing mass over the index finger and palm without any bony destruction; (B) lateral view of the right hand showing mass over the index finger without any bony destruction

A 30-year-old man came to our outpatient department with a mass over his right index finger for six months, which was insidious and progressive in nature, associated with pain and restricted finger movements. On clinical examination, a 3x2 cm swelling firm in consistency was noticed over the right index finger over the middle phalanx, extending toward the distal interphalangeal joint, and a small 1x1 cm swelling was palpable in the palmer area near the thumb. The X-ray revealed a mass on the palmar aspect of the index finger and palm without any bone destruction (Figure 1B).

An outside MRI impression gave a differential diagnosis of soft tissue hemangioma, synovial tumor, or peripheral nerve sheath tumor. However, as the surgeon could not make a sure diagnosis from imaging, a decision was made to go for the excision of the tumor in the index finger and palm. The excised specimen was sent for histopathological examination to confirm the final diagnosis. Grossly, the specimen was sent as three small, gray-white, soft-to-firm, well-demarcated tissue pieces ranging from 0.1 to 3 cm.

Microscopy

**Figure 2:**
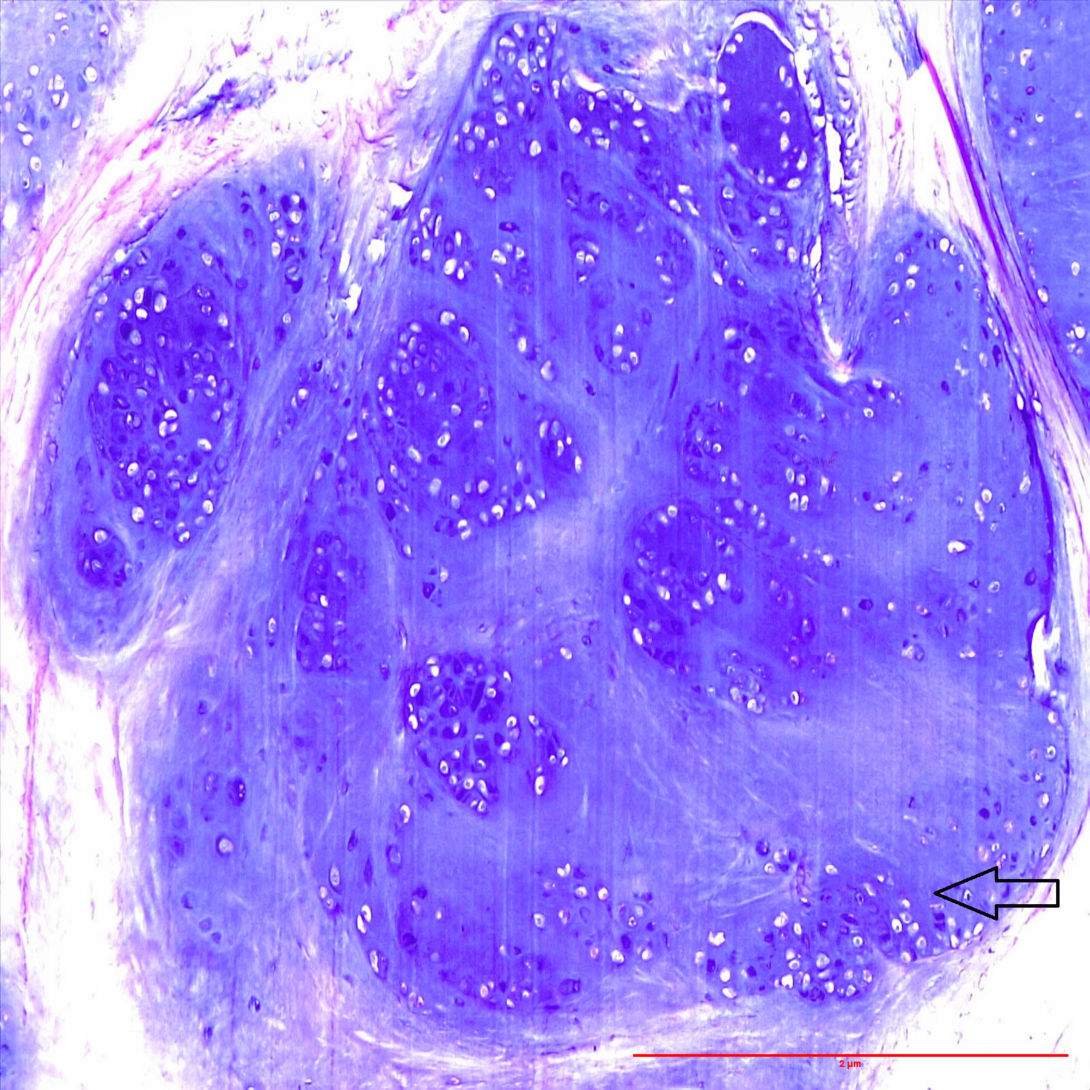
High-power view: Hematoxylin and eosin (H&E)-stained slides show mature hyaline cartilage with mononuclear chondrocytes without evidence of cytological atypia/mitosis (H&E: 40x)

**Figure 3:**
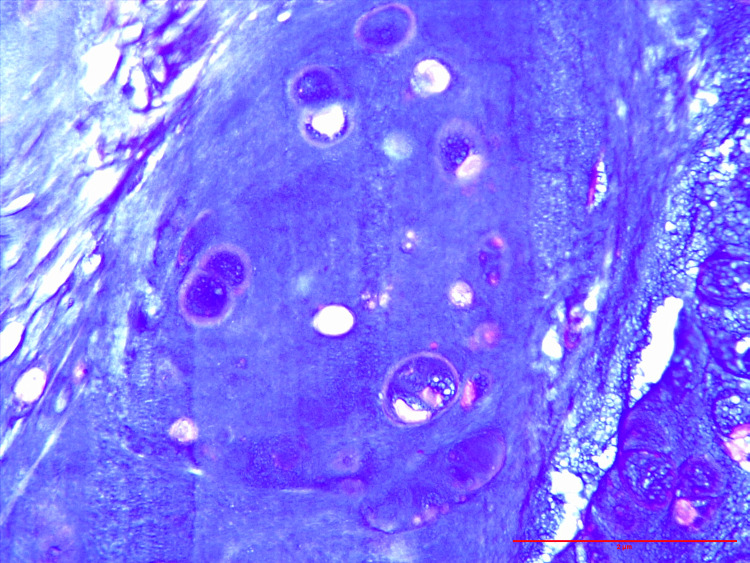
High-power view: Hematoxylin and eosin (H&E)-stained slides show chondrocytes with uniform round nuclei without evidence of atypia or mitosis (H&E: 40x)

The section shows a tumor composed of lobules and islands of mature benign hyaline cartilage with well-vascularized stroma and areas of calcification. The cartilage is consistent with bland-appearing chondrocytes in the lacuni without cytological atypia (Figure 2). The chondrocytes show uniform round nuclei without any atypia (Figure 3). The lesion on the palm also shows the same histological findings. Hence, the final diagnosis of extraskeletal chondromatosis was made. Postoperatively, the patient is doing well; there are no other complaints.

## Discussion

The extraskeletal chondroma is a benign cartilaginous tumor. It mostly manifests in the soft tissues. Rather than coming from fully developed osseous or cartilaginous tissue, it is believed to originate from the fibrous stroma of soft tissues. Tissue chondromas frequently have FN1 gene rearrangements linked to them [6]. Frequent follow-up is advised because, although the local recurrence incidence rate of 15-18% is relatively unusual, there is a possibility that it resembles the tumor after recurrence. Re-excision is the best treatment for local recurrence. A well-defined soft tissue mass with typical central calcifications or areas of ossification is revealed by an extraskeletal chondroma on a CT scan. To rule out a malignant soft tissue tumor, tissue sampling should be done on a soft tissue mass that shows no imaging abnormalities. It usually presents as a nodular soft tissue mass that slowly enlarges without pain. The amount of calcification and water content in the soft tissue chondroma affects the signal intensity of the T2-weighted image (T2WI) in MRI. The confusing fact is that sometimes the cartilaginous cells have an acidophilic cytoplasm simulating that of a histiocyte and sometimes a vacuolated appearance reminiscent of a lipoblast. Benign tumors like calcifying aponeurotic fibroma (CAF), tumoral calcinosis (TC), giant cell tumor of the tendon sheath (GCT), and synovial chondromatosis (SC) need to be differentiated from extraskeletal chondroma [7,8]. TC shows similarities with calcified extraskeletal chondroma (ESC), but it lacks cartilage. CAF occurs in young patients, commonly over the hands, with foci of cartilaginous metaplasia in a dense fibromatous background. GCT shows a uniform cellular pattern with a rarity of metaplastic cartilage. Also, these cases show cellular pleomorphism and myxoid changes, which should be differentiated from extraskeletal myxoid chondrosarcomas (ESMCSs), which are malignant tumors that can metastasize. ESMC is less differentiated at the periphery of the tumor, whereas the chondrocytes in ESCs have better differentiation in the peripheral region of the tumor. The cellularity, nuclear size, mitotic rate, and frequency of lacunae with multiple nuclei are among the features that determine the pathological classification of mesenchymal chondrosarcoma (MC) grades I and II. Spindle cells are usually present in the mesenchymal subtype. Within the myxoid matrix, a series of spherical cells make up the myxoid type. The mesenchymal chondrosarcoma reveals a dimorphic histologic appearance in which malignant hyaline cartilage is combined with mesenchymal tissue [9]. However, our case shows a typical pattern of chondroma without atypia or mitosis, hence the diagnosis of ESC. An aggressive osseous lesion with minimal chondroid matrix mineralization and an intermediate signal on T2WI (lower than that of conventional chondrosarcoma) with more dramatic enhancement than expected with conventional chondrosarcoma imply the diagnosis of mesenchymal chondrosarcoma [10]. The World Health Organization's (WHO) fifth edition categorization of soft tissue and bone cancers provides diagnostic criteria that include limited chondroid cell atypia and mitotic activity, a hyaline or myxoid matrix, and soft tissue tumors with lobules of well-defined cartilage [11]. Marginal tumor resection is the treatment of choice. This has a comparatively high local recurrence rate. It must be removed completely, including the capsule, to prevent any remnants from reoccurring.

## Conclusions

Extraskeletal chondroma of the index finger is a rare occurrence. As the histomorphological features are similar to those of other tumors, like low-grade chondrosarcoma, the alarming sign of differentiation becomes a must. Awareness of the patterns of internal calcification is essential to narrowing the diagnosis to a cartilaginous tumor. Hence, the histopathological assessment of the tissue is of great importance.
